# Fused filament fabrication of polyetheretherketone in vacuum: the influence of high vacuum on layer adhesion in z-orientation

**DOI:** 10.1007/s40964-024-00897-2

**Published:** 2024-12-08

**Authors:** Marina Kühn-Kauffeldt, Marvin Kühn, Christoph Mittermeier, Josef Kiendl

**Affiliations:** 1 Institute of Electrical Energy Systems, Universität der Bundeswehr München, Werner-Heisenberg-Weg 39, Neubiberg, 85579 Germany; 2 Institute of Engineering Mechanics and Structural Analysis, Universität der Bundeswehr München, Werner-Heisenberg-Weg 39, Neubiberg, 85579 Germany

**Keywords:** FFF, PEEK, Vacuum, Pressure dependence, Performance

## Abstract

The application of fused filament fabrication (FFF) in vacuum changes the heat transfer of the process. This work investigates the influence of the working ambient pressure conditions in FFF-based 3D printing of polyetheretherketone (PEEK) specimens, and its impact on the resulting part strength. Layer adhesion drastically improves with decreasing pressure, maximum layer adhesion is reached for ambient pressure below $10^{-3}$ mbar. We show that simple and low-cost vacuum equipment is sufficient to achieve such pressure conditions, making this process interesting for the general processing of high-temperature polymers using FFF.

## Introduction

Fused filament fabrication (FFF), a cornerstone of additive manufacturing technologies, offers transformative capabilities in creating three-dimensional objects layer by layer from thermoplastic materials. While traditionally operated under standard atmospheric pressure ($10^{3} \textrm{mbar}$), the integration of FFF within a vacuum environment introduces a novel paradigm in manufacturing, unlocking unprecedented material properties and application possibilities.

Out-of-Earth manufacturing and assembly technologies present significant advantages, including the potential for virtually unlimited overall volume, enhanced design flexibility, and the capability to upgrade and repair existing spacecraft and satellites, thereby fostering sustainable space utilization [1]. These technologies enable the construction of structures tailored to meet the specific physical and functional requirements of various environments, such as Low Earth Orbit, the Moon, Mars, and beyond. Consequently, only raw materials need to be transported to the off-Earth manufacturing site, a process that can be executed more efficiently due to the compact and less fragile nature of the payload. Additionally, Out-of-Earth manufacturing facilitates recycling and the utilization of local resources. The adoption of FFF in vacuum conditions is underscored by its pivotal role in out-of-earth manufacturing [2] and its compatibility with hybrid material deposition techniques [3]. This refined approach offers critical advantages for space exploration, facilitating in-situ manufacturing capabilities that are crucial for long-duration missions or manufacturing of large structures.

Furthermore, processing in a vacuum significantly reduces the interaction of materials with atmospheric gases, resulting in components with superior mechanical properties and reliability. Moreover the combination of FFF with other vacuum-based material deposition processes, such as electron beam melting or sputtering could also foster the development of composite materials and complex multi-material structures. Additive manufacturing is already being considered for the production of medical devices and implants [4]. Here, the vacuum environment with its naturally provided sterile setting might considerably rise the interest in this manufacturing technique.

In previous works, a general feasibility of FFF manufacturing in vacuum was already demonstrated for different materials such as polycarbonate (PC) [5] and polyetheretherketone (PEEK) [6]. In these papers a working pressure of around 1 mbar was used. Spicer et al. have extensively investigated the extrusion of polylactic acid (PLA), polyethylene terephthalate glycol (PETG), acrylonitrile butadiene styrene (ABS), polyetherketoneketone (PEKK) and polyetherimide (PEI) at a pressure of $10^{-5}\,\textrm{mbar}$ [7, 8]. Moreover, processing of PEEK under pressures of around $10^{-4}\,\textrm{mbar}$ [3] and processing of as obtained and recycled PEEK, PEKK, and PEI at a pressure of around $8 \times 10^{-5}\,\textrm{mbar}$ [9] was demonstrated. However, most of the work demonstrated the feasibility and merely investigated the mechanical properties of such produced specimens. Influence of high vacuum as it prevails on the moon was only theoretically considered in [10]. The influence of varying pressure up to the magnitude of high vacuum on the FFF process and the resulting properties of the printed object were not investigated experimentally so far. The objective of this work ties in with previous research by investigating the influence of different ambient pressure levels on the resulting z-layer adhesion. Ambient pressure levels below $10^{-1}\,\textrm{mbar}$ strongly influence the cooling process due to the reduced convectional heat losses. Convective losses, being the main cooling mechanism in the standard atmospheric pressure, are absent. Hence the cooling process is mainly driven by heat conduction into the heat bed and the radiative losses [11, 12]. It can be assumed, that the heat transport near the print bed differs from that one at a certain height. Therefore, the samples are printed over the entire available height to have the zone tested later at a height with homogeneous heat transport. To investigate the z-layer adhesion at a substantial distance from the build plate we chose to produce a specimen in spiral vase mode. This mode creates a continuous single outline container, gradually increasing the z-height.

There are various approaches in the literature for determining the adhesive strength of FFF printed specimens in the z-direction. In [13], hollow boxes with walls of equal length that are one strand thick are printed. A mini laser and a die cutter were used to cut out the tensile test specimens. Both cutting methods led to identical results. Another approach is the three-point bending test described in [14]. The bending specimens are tubes with a rectangular cross-section and a wall thickness of four strands. In [6] tensile specimens, which are made of PEEK, are not cut but printed directly in the correct shape with a thickness of four strands.

In this contribution, a bending test device is used since the specimens printed at normal pressure have low adhesion in the z-direction and are therefore not suitable for cutting. The tubular specimens with a circular cross-section are printed in spiral mode. Due to the resulting single-strand thin wall, the bending test device is modified.

## Materials and Methods

### FFF system

Specimens were fabricated using a custom-made FFF system with a build volume of 100 mm $\times$ 100 mm $\times$ 65 mm (Fig. 12B). The kinematics were implemented as a belt-driven (X- and Y-axis) rectilinear Cartesian system powered by Nema 17 vacuum-rated motors (Lin Engineering, USA). The Z-axis consists of a single, lead screw-driven linear rail carrier onto which the X-axis was mounted. This configuration was chosen to reduce the number of parts that could contaminate the vacuum. Here, a full metal hot end was chosen to be able to print high-temperature polymers. It uses an E3Dv6 copper heater block, including a heater cartridge, and a PT100 temperature sensor, an E3D v6 1.75 mm stainless steel heat break and a custom-made water-cooled cold end made of aluminum. The extruder is a dual-gear bowden extruder (Micro Swiss, USA). The maximum hot end temperature is 450$^\circ$ C. All the mechanical components (e.g., linear rails and spindles) were chosen to be lubricant-free or greased with vacuum-rated lubricant to minimize outgassing in the vacuum. The motors and the extruder were actively water-cooled (H2O Komplettmodul, Innovatek, Germany). For all prints, a 0.6 mm stainless steel nozzle was used. The aluminum print bed is custom-made and uses a high-power resistor (LPR 50 W @ 12 $\Omega$, Arcol) for heat generation and a Borosilicate glass as a print surface. The maximum heat bed temperature is 180 $^\circ$C. The printer is controlled by a FYSETC SPIDER v.1.2 board equipped with TMC2009 motor drivers and uses KLIPPER firmware in combination with a Raspberry Pi computer. Furthermore, a BL-Touch (Antctlabs, South Korea) is used as a bed leveling sensor.

### Vacuum setup

The FFF system was positioned inside a stainless steel vacuum chamber (inner dimensions 30 cm $\times$ 30 cm $\times$ 30 cm) equipped with an acrylic door as a viewport (Fig. 12A). For vacuum generation, a HiPace 80 Turbo Pump together with a Duo 5 m backing pump and a DCU02 control unit were used (Pfeiffer Vacuum, Germany). The pressure was monitored via a PKR 251 wide-range pressure sensor (Pfeiffer Vacuum, Germany). The FFF hardware was electrically connected via standard electrical feedthroughs to the electronic boards that were located outside the vacuum chamber. For the water-cooling circuit, straight screw connections and fluororubber (FKM) tubings were used to link the serially arranged heat sinks in front of the hotend and motors to a radiator. In this way, the outgassing from the FFF cooling system was minimized, while giving the kinematic system the necessary flexibility. Vacuum setup entailing the FFF system could reach pressure in the range of 10$^{-5}$ mbar without the operation of the extruder unit.

### Specimen fabrication

A PEEK filament with a 1.75 mm diameter (PEEK, INFINAM PEEK 9359 F, Evonik, Germany) was used for all test specimens. The filament spool was dried at 200 $^\circ$C for 120 min before it was used in the printing process. Afterwards, the spool was placed into the vacuum chamber as shown in Fig. 12C. The high vacuum protects the filament from water absorption during the manufacturing process, which makes additional drying of the filament between the prints unnecessary. The test specimen is a single-walled tube with an inner diameter of 10 mm and a length of 60 mm. The corresponding G-codes were generated with Prusa Slicer v.2.6.3 software and the spiral vase mode enabled. Three identical test specimens were printed during one vacuum cycle using the multiple print option in the Prusa software. For better bed adhesion a brim together with a build plate coating (Nano Polymer Adhesive, VISIONMINER, USA) was used.

To select appropriate process parameters for printing under varying pressure conditions, it is essential to identify settings that yield optically comparable results. For extrusion under standard atmospheric conditions, the nozzle temperature must be significantly higher than the melting point (343 $^\circ$C) to offset convective heat losses. In this study, reliable extrusion was achieved at a nozzle temperature of 400 $^\circ$C at the pressure of 10$^{3}$ mbar, which was used for the following experimental series.

The second parameter that required optimization was the printing speed. When the extruded material is exposed to atmospheric conditions, it cools down and solidifies rapidly. However, this process is markedly slower in a vacuum environment. Preliminary tests were conducted to assess different printing speeds ranging from 1 mm s$^{-1}$ to 10 mm s$^{-1}$ to identify the optimal extrusion velocity at a pressure of 10$^{-4}$ mbar. It was observed that at a speed of 5 mm s$^{-1}$, the material from the previous layer had fully solidified when the new layer was deposited on it, indicating a suitable balance for the specific specimen geometry employed in this study. Hence the printing speed was set to 5 mm s$^{-1}$ for all specimens.

The FFF process speed and extruder parameters used in this study are summarized in Table 1. For the investigation of the influence of the ambient pressure on the part strength, sets of 3 specimens were printed at 5 different pressure settings: 10$^{-3}$ mbar, 10$^{-2}$ mbar, 2 $\times$ 10$^{1}$ mbar respectively.

**Table 1:** Printing parameters

Parameter	Values
Nozzle temperature, first layer [$^\circ$C]	410
Nozzle temperature, standard [$^\circ$C]	400
Bed temperature [$^\circ$C]	150
Chamber temperature [$^\circ$C]	r.t
Printing speed [mm s$^{-1}$]	5
Layer height [mm]	0.25
Extrusion width [mm]	0.8

### Bending test device

**Fig. 1:**
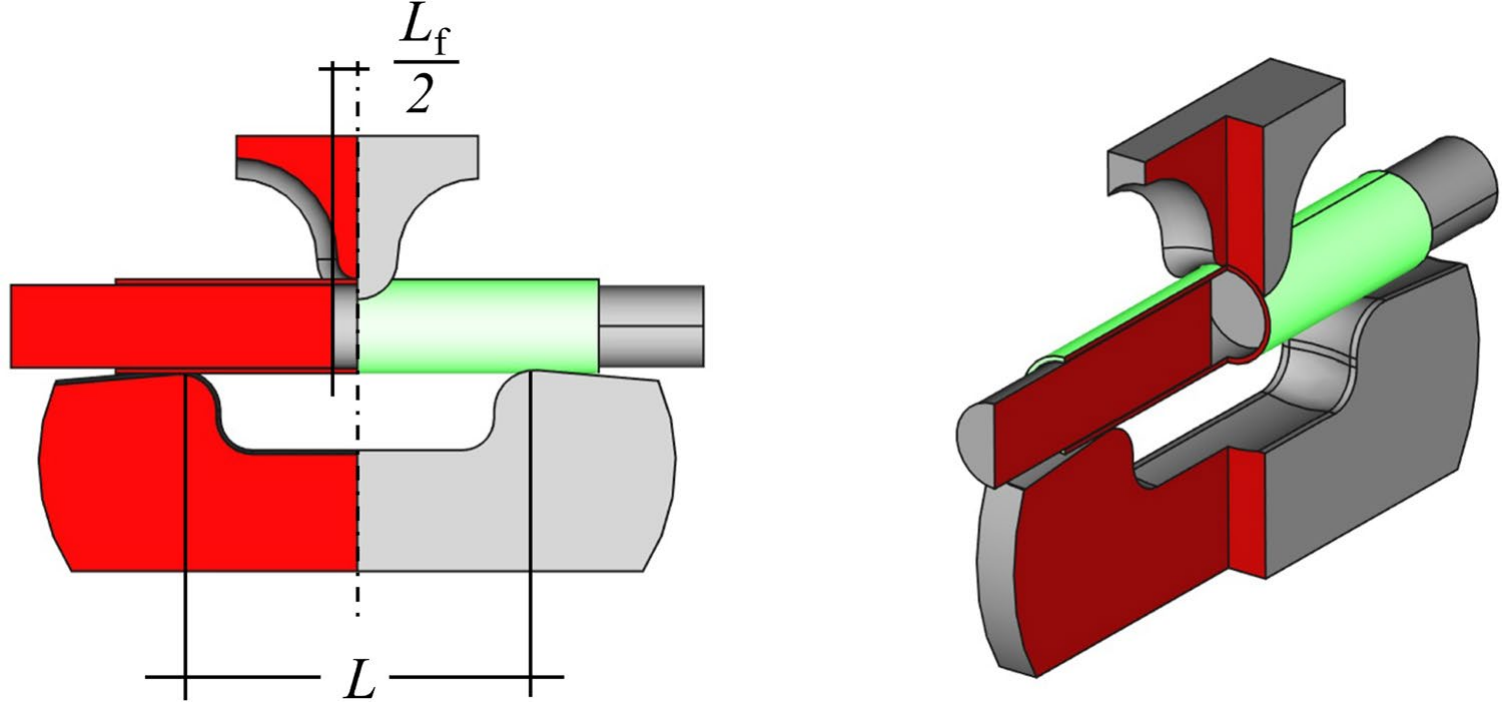
Front and three-dimensional view of the CAD model of the test device (with a quarter cut out, see red areas), the fin at the top, the specimen (green) with the two support cylinders in the middle and the two abutments at the bottom

The design of the used test device is based on the standard DIN EN ISO 178. As shown in Fig. 1, the printed specimen is pressed by a fin from above against two abutments. Thereby a span length of $L =$ 42 mm and a displacement rate of 0.03 mm s$^{-1}$ is used. The tests were performed at ambient temperature ( 20 $^\circ$C) on the Inspekt universal testing machine (Hegewald and Peschke Meß- und Prüftechnik GmbH, Germany) with a 5 kN load cell. Testing was stopped when the load had reduced by 20 % from the maximum. Each of the specimen configuration was tested three times to ensure repeatability.

**Fig. 2:**
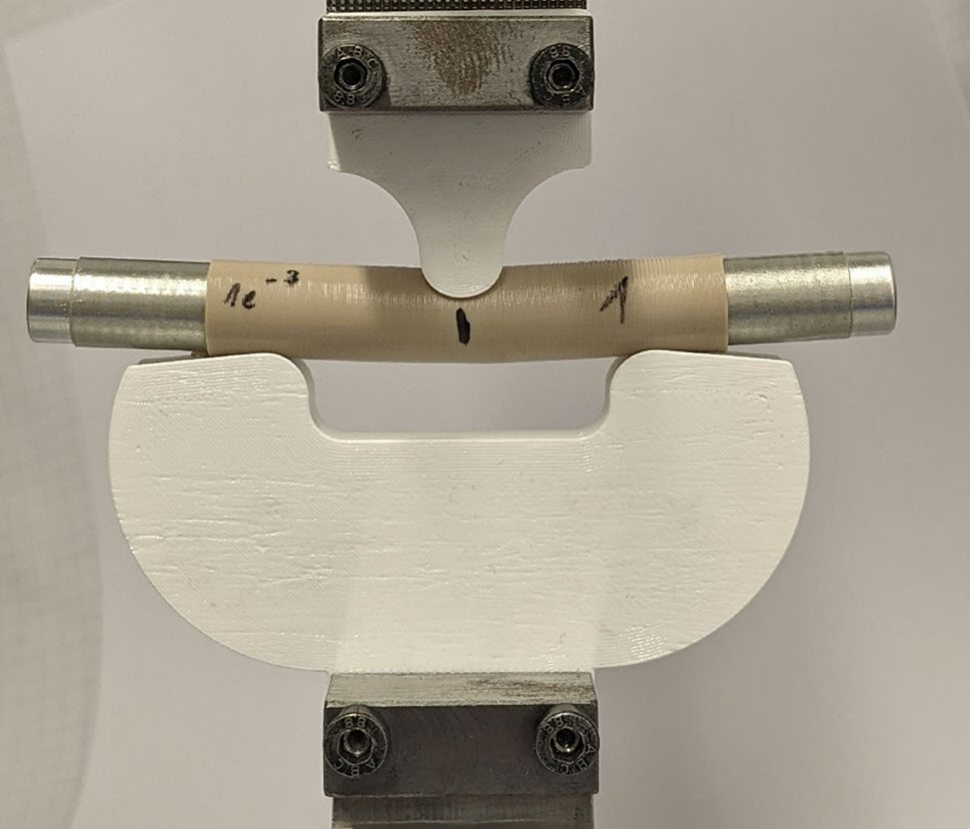
The test device is in operation shortly before the specimen breaks. Here an additional transparent tape is used as to mark the position of the aluminum cylinder in the specimen

In Fig. 1 two aluminum cylinders can be also seen that fit into the inner diameter of the two ends of the specimen. They prevent its thin-walled tube shape from buckling. These cylinders are not connected in the middle of the specimen under the fin and thus leave $L_f =$12 mm of the span length open. It is also shown in Fig. 1 that the fin and the abutments are concavely curved in the plane of the specimen tube cross-section. Firstly, this distributes the pressure load under the fin on a line rather than a point, which further reduces the risk of buckling. Secondly, the specimen automatically aligns itself correctly, when inserted and does not roll or slip away during the test. In Fig. 2 the device can be seen in operation.

**Fig. 3:**
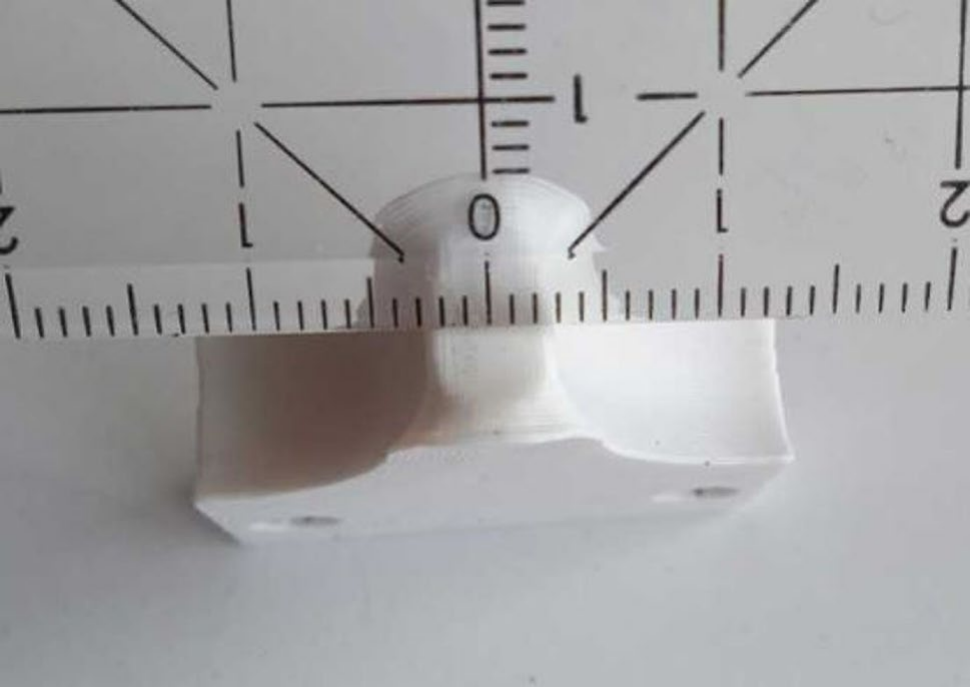
Detail of the permanent symmetric deformation of the fin after the first loading in the blind test, which leads to two contact lines with a distance of $L_s =$5 mm between them

It is printed on a standard Prusa FFF printer with glycol-modified Polyethyleneterephthalate (PETG) filament. The flexibility of the device was determined by several blind tests before and after the tests on the specimens. In the blind test, an aluminum cylinder, which is orders of magnitude stiffer than the test specimens and has the same outer diameter, was loaded with a force that was two times higher than the maximum force when testing the specimens. The first loading leaves a permanent symmetrical deformation in the fin, which can be seen in Fig. 3. Afterwards, the fin was moved up until the force was almost zero. All following repetitions of the blind tests lead to identical force-displacement curves and no additional permanent deformations. In Fig. 3 the two outside border lines of the permanent deformation with the distance $L_s$ = 5 mm between them can be also seen. In the bent configuration, the fin touches the sample on these two lines. This turns the device originally intended for a three-point bending into a four-point bending device.

In the later evaluation, the force-dependent displacements of the blind test are subtracted from the displacements measured in the specimens test to compensate for the flexibility of the device. When considering the test as a four-point bending test, the equations used for calculating the stress $\sigma _f$ and the strain $\epsilon _f$ on the outer surface of the tensile side within the two contact lines of the fin are:

1 $$\begin{aligned} \sigma _f&=\frac{F(L-L_s)}{4w_b} \end{aligned}$$

2 $$\begin{aligned} \epsilon _f&=-\frac{sD}{{\frac{1}{12}(L-L_s)^2+\frac{1}{4}(L_s^2-L^2)+\frac{1}{6}\frac{(L-L_f)^3}{L-L_s}}}. \end{aligned}$$

**Fig. 4:**
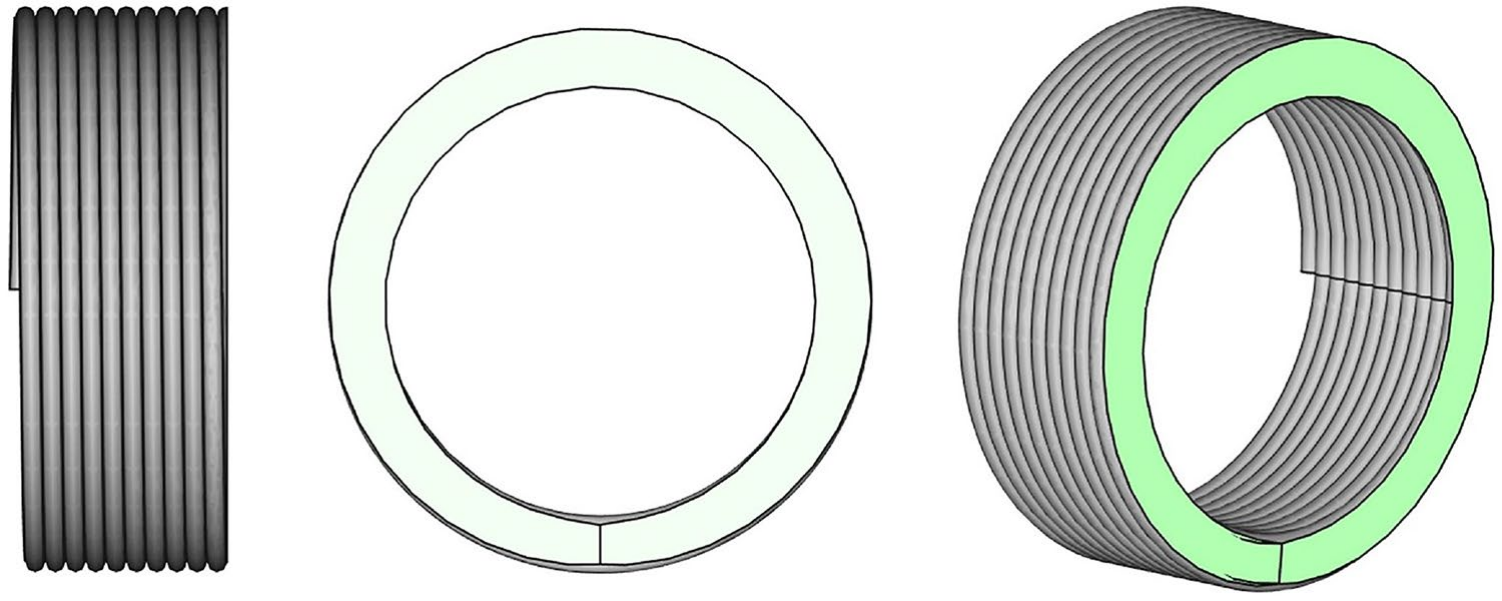
Principal sketch (side-, front- and three-dimensional view) of the unusual shape of the cross-section (green cut) resulting from the spiral specimen geometry

Therein, *F* is the acting force, *D* the diameter of the specimen, *L* =42 mm the length between the two abutments (as shown in Fig. 1), $L_s$ = 5 mm the distance between the two contact lines of the fin (as shown in Fig. 3), $w_b$ the moment of bending resistance, *s* the deflection of the specimen at the contact to the fin and $L_f$ = 12 mm is the distance between the support cylinder (as shown in Fig. 1). Equation (1) is based on the 4-point bending moment curve and Eq. (2) is derived from the bending differential equation. The boundary conditions for Eq. (2) are defined by the 4-point bending moment curve and the support cylinders are assumed to be infinitely rigid. For a tube specimen with a cylindrical cross-section the moment of bending resistance is calculated by:

3 $$\begin{aligned} w_b =\frac{\pi }{32}\bigg (\frac{\big (D^4-(D-2t)^4\big )}{D}\bigg ), \end{aligned}$$

where *t* is the wall thickness of the specimen. Figure 4 shows that the spiral geometry of the specimen results in a change of the thickness along the circular line of the cross-section.

**Table 2:** Geometrical parameters of the printed specimens

P [mbar]	$10^{-4}$		$10^{-3}$		2 $\times 10^{-1}$		2		10$^{3}$	
Nr./ Dimensions [mm]	D	t	D	t	D	t	D	t	D	t
1	11.21	0.63	11.21	0.61	11.23	0.63	11.23	0.64	11.36	0.61
2	11.22	0.63	11.14	0.62	11.23	0.63	11.29	0.64	11.38	0.62
3	11.13	0.64	11.12	0.62	11.18	0.63	11.32	0.63	11.36	0.64
$\mu$ [mm]	11.19	0.63	11.16	0.62	11.21	0.63	11.28	0.64	11.37	0.62

Therefore, the stress distribution in the orthogonal direction to the cross-section is inhomogeneous when considered in detail. As this detailed consideration is not necessary for the comparison between the different pressures, the definition in equation eq. (3) is used. For this purpose, *D* and *t* are measured with a caliper gauge (0.01 mm precision), see table 2.

### Specimen characterisation

To investigate the layer strucutre of the specimens in z-direction, micro-computed tomography ($\mu$CT, Bruker SkyScan micro-CT 117, USA) images have been acquired with a resolution of up to 5 $\upmu$m.

The fracture surface was analyzed by means of a laser scanning microscope (LSM, VK-X 3000, Keyence, Neu-Isenburg, Germany). To capture the surface of the whole specimen the image stitching module of the VK-X3000 Software provided by Keyence was used.

X-ray diffraction is performed to calculate the degree of crystallinity of printed specimens using Bruker D8 Discover, (Bruker, USA). The diffraction angle $2\Theta$ is ranged from 10$^\circ$ to 60$^\circ$ with an increment of 0.05$^\circ$. The diffractometer system uses Cr tube as an X-ray source with an intensity of 40 mA and a tension of 45 kV in the spot mode to account for the surface roughness of the specimens. Here a polycapillary lens and a collimator with an opening of 1 mm were positioned in the primary beam path.

## Results and discussion

**Fig. 5:**
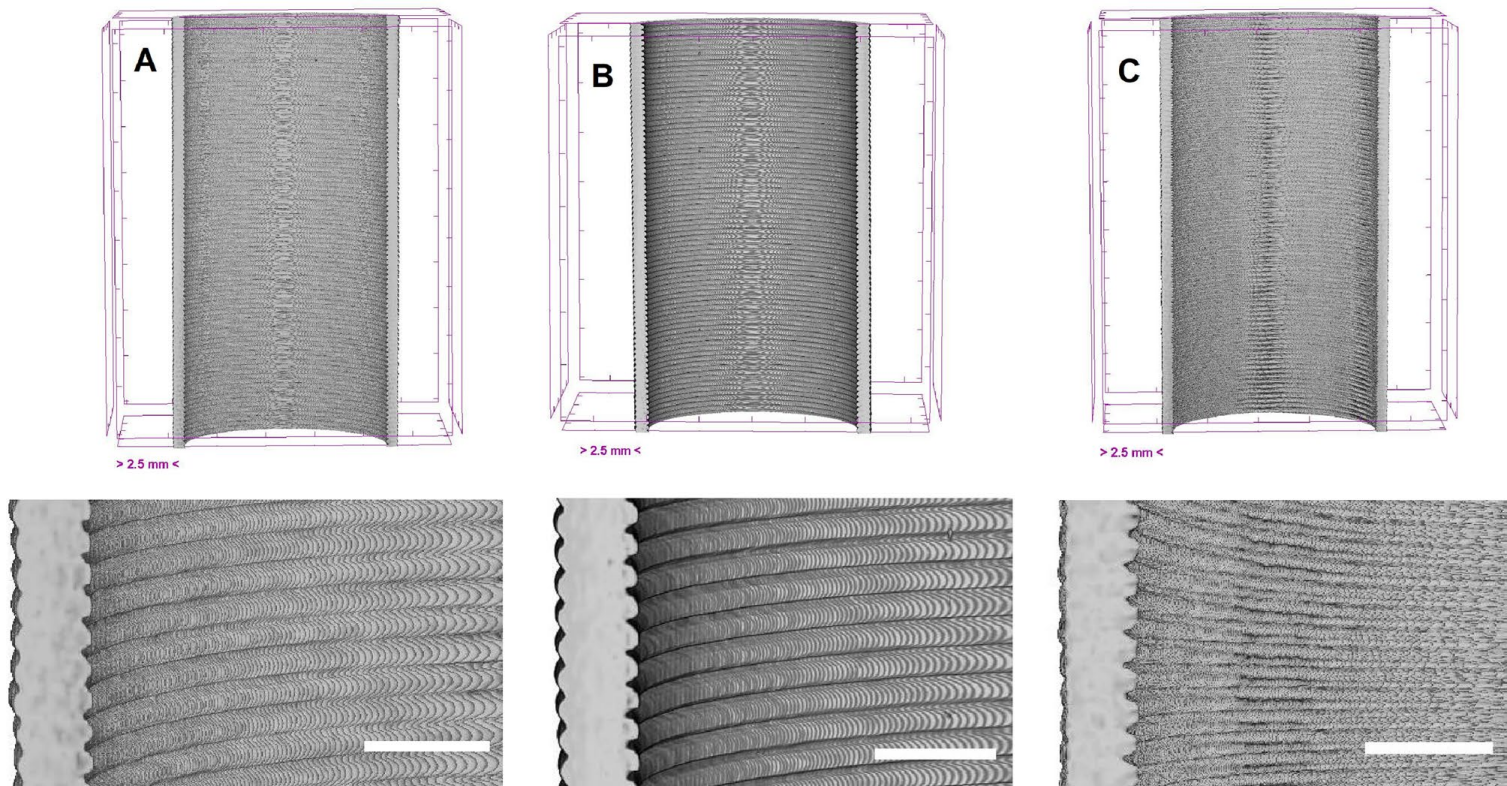
Overview crosscut (top) and close-up (bottom) of the volumetric $\mu$CT scan rendering of specimens printed at (**A**) $10^{3}$ mbar, (**B**) 2 mbar and (**C**) $10^{-3}$ mbar. The scale in the overview images corresponds to 2.5 mm and to 1 mm in the close-up views

This study evaluated the impact of different pressure conditions on the z-layer adhesion of specimens using the bending test described above. For each pressure, three samples were printed and tested. Figure 5 shows crosscut view of volumetric renderings of $\mu$CT scans with a close-up view below each crosscut. Here specimens were scanned prior to bending tests for three different ambient pressures. All three samples show homogeneous layer structure without voids. While the close-ups of specimens printed at 10$^3$ mbar and 2 mbar look alike, the layers of the specimen printed at 10$^{-3}$ mbar change in shape, which might origin from a longer solidification time of each layer.

**Fig. 6:**
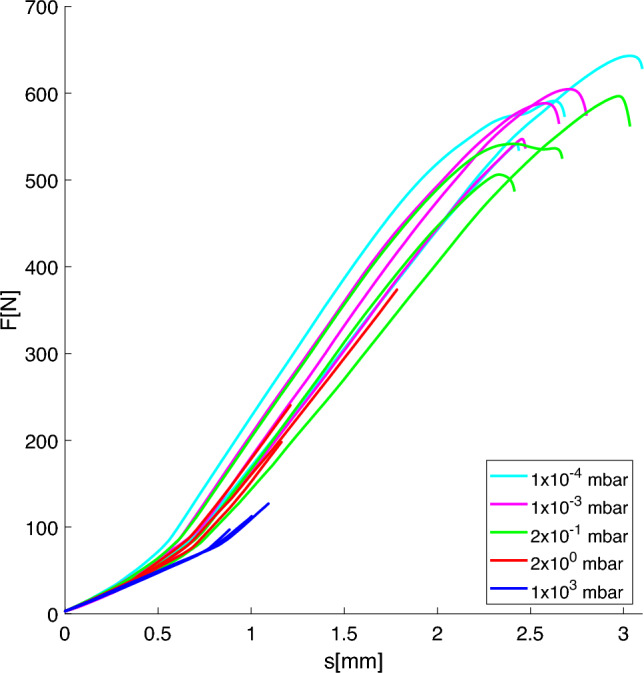
Force-displacement curves of the bending test

**Fig. 7:**
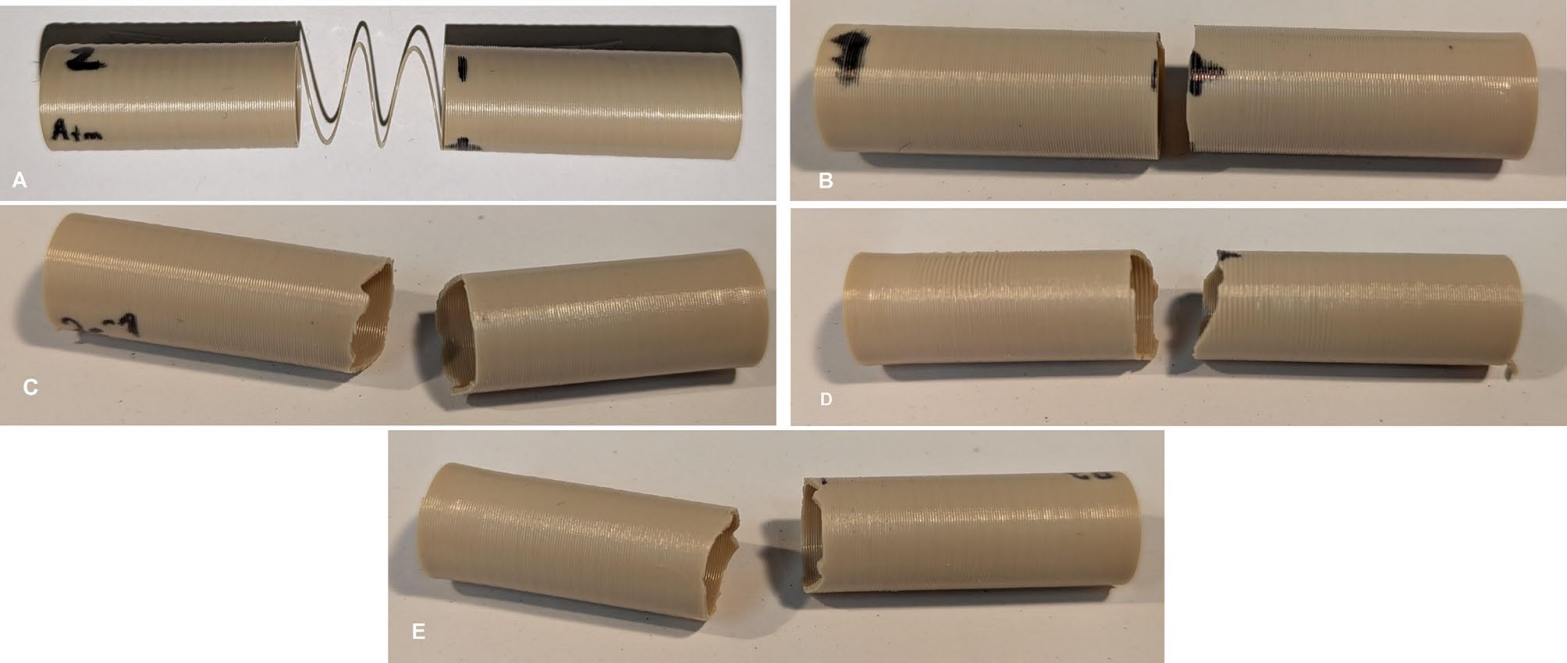
Specimen printed at $10^{3}$ mbar (**A**), 2 mbar (**B**), $2 \times 10^{1}$ mbar (**C**), $10^{-3}$ mbar (**D**) and $10^{-4}$ mbar (**E**) after the bending test

Table 2 reports the measured geometrical properties of each specimen and the mean value $\mu$ for each pressure. It can be noted, that a diameter deviation of around 2 % can be found when comparing the average dimensions of specimens printed at $10^{-4}$ mbar and $10^{3}$ mbar. A possible explanation is that the cooling process is much faster at regular atmospheric conditions than in high vacuum. Here, the polymer chains have time to rearrange and shrink due to the way more retarded cooling mechanism. Figure 6 shows the resulting force-displacement diagram. Here it can be seen that the maximum displacement and force significantly rise with decreasing pressure set during the manufacturing process. Figure 7 shows photographs of five specimens after the bending test. While the specimen printed in standard atmospheric conditions shows layer delamination at the fracture, it is not the case for the specimen printed at 2 $\times 10^{-1}$ mbar, $10^{-3}$ mbar and $10^{-4}$ mbar. Here, the fracture extends over several layers, revealing another evidence of an improved layer adhesion. The specimen printed at 2 mbar has a more straight fracture which might indicate slightly reduced tensile strength.

**Fig. 8:**
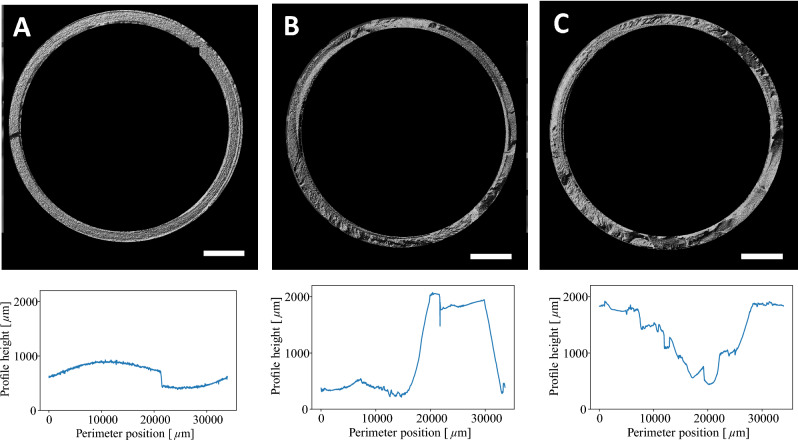
Fractography images displayed in circular differential interference contrast for (**A**) $10^{3}$ mbar, (**B**) 2mbar and (**C**) $10^{-3}$ mbar together with height profile of the specimen recorded along the perimeter of each probe shown below each image. Scale bar corresponds to 2000 $\upmu$m

In addition. fractography images of the specimens printed (A) at 10$^{3}$ mbar, (B) at 2 mbar and (C) at 10$^{-3}$ mbar are shown in Fig. 8. The circular differential interference contrast view is used to emphasize structural irregularities in different focal plains and light contrast. For the specimens printed in vacuum the fractography images and the evaluated perimeter height profiles highlight the irregular break surface. However, the specimens printed at $10^{3}$ mbar which break along the layer interface have a relatively smooth surface, which is also highlighted by the profile. Since it was necessary to cut the layer strand of this specimen for imaging, a step can be seen in the profile at the location of the cut.

**Fig. 9:**
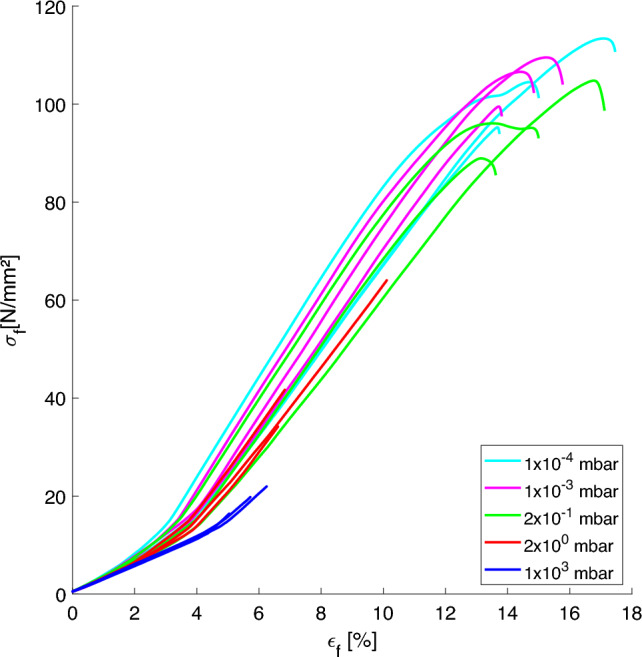
Stress–strain curves of the bending test

Equations (1) and (2) and the values from table 2 were used to calculate the stress–strain diagrams shown in Fig. 9. It should be considered, that these calculations are based on ideal assumptions. Moreover, flexural tests are known to achieve higher stress yields compared to tensile tests [15]. This should be taken into account when comparing these results to other data.

According to the data sheet [16], the stress yield of the PEEK filament value of 90 MPa lies below the measured flexural strength of the specimens in this work. Since the filament strength was determined according to the ISO 527 tensile test standard, exceeding flexural strength values can be expected. When comparing the results with other flexural tests conducted for PEEK specimen, maximal values of up to 142 MPa are reported for specimens bent in the direction of the filament strand [17]. For the z-printing orientation also investigated in this work values in the range between 16 MPa to 30 MPa are reported in [17, 18]. The average flexural strength of 19 MPa calculated for 10$^{3}$ mbar specimens is comparable to these values. The values for specimens printed at lower ambient pressure greatly exceed the reported values, which indicates a superior layer adhesion.

The strain at break for specimens printed at 103 mbar is in the range of 5 %, which is slightly higher than values reported for vertically printed probes [17, 18]. The specimens printed at lower pressure starting form 2 $\times 10^{-1}$ mbar deliver average values of 15 % which is comparable to strain at break values reported for specimens bent in the strand direction [17]. This again indicates, that z-inter-layer material properties established at lower ambient pressure come close to the filament material properties.

**Fig. 10:**
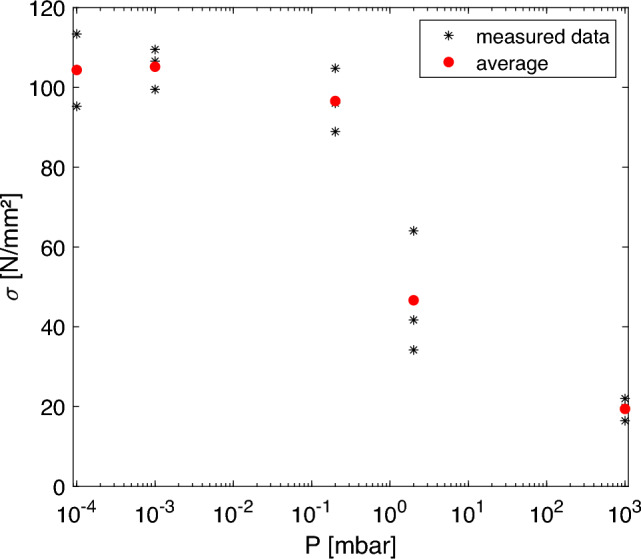
Maximal flexural strength of the bending test reached at different ambient pressure during printing. Black stars denote the measured data together with the calculated average (red circle)

Figure 10 provides an overview of the flexural strength trend as the ambient pressure rises. Here, specimens printed at 2 $\times 10^{-1}$ mbar already deliver flexural strength values close to the maximum. The latter is reached for $10^{-3}$ mbar to $10^{-4}$ mbar.

**Fig. 11:**
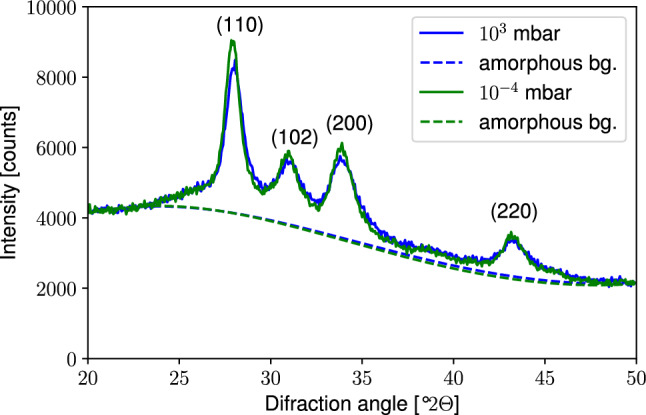
Diffractogram of PEEK specimens printed at 10$^{3}$ mbar (blue) and 10$^{-4}$ mbar (green) together estimated amorphous background

In addition, the crystallinity of specimens was accesses by means of XRD after the bending tests. Figure 11 visualizes diffractogrms of specimens printed at $10^{3}$ mbar and 10$^{-4}$ mbar, where most significant deviations could be observed. The most prominent difference can be seen for diffraction peaks (110) and (200), where the signal from specimen printed in vacuum exceeds signal of that printed at $10^{3}$ mbar.

**Fig. 13:**
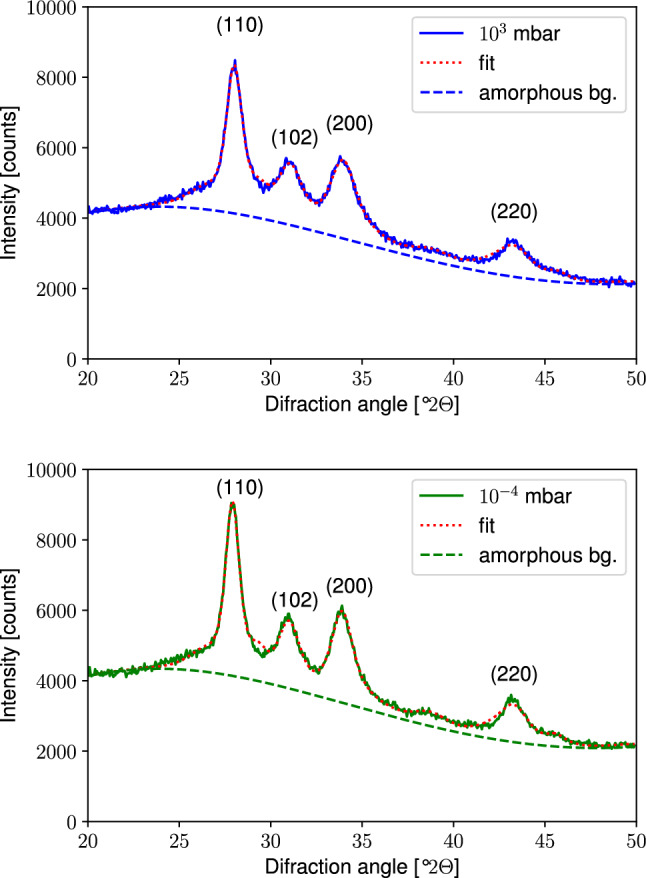
Diffractogram of PEEK specimens printed at 1 $\times 10^{3}$ mbar (blue,top) and 1 $\times 10^{-4}$ mbar (green,bottom) together estimated amorphous background. Here total spectral fits produced by Rietveld refinement of PEEK crystalline structure under consideration of instrumental function are shown in red. From the fits the background estimation was extracted and used for further crystallinity calculation

The crystallinity was evaluated by calculation of the $\chi _{PEEK}=\frac{I_{total}-I_{back}}{I_{total}}$ [19] with $I_{total}$ the integral over the diffraction angle of the total measured signal and $I_{back}$ the integral over the estimated background. The latter was estimated using Retvield refinement procedure implemented in the Profex 5.2 software [20]. The total fitted spectra from which the background was extracted are shown in the appendix (Fig. 13). Here $\chi _{PEEK,10^3}=$(17.23 ± 0.16) % was calculated for specimens printed at 10$^{3}$ mbar and $\chi _{PEEK,10^{-4}}=$(17.45 ± 0.26) % for those at $10^{-4}$ mbar.

The order of magnitude of the crystallinity is comparable to values obtained from melted state measured by XRD in [19]. In this study, the crystallinity reached a similar level in both investigated cases. Hence it can be concluded, that the superior flexural strength of specimens printed in a vacuum does not originate from the degree of crystallinity but more likely from improved inter-layer bonding for the specific specimen geometry.

The fact that a superior z-layer adhesion can already be reached by a pressure of 10$^{-3}$ mbar also leads to technical advantages for the vacuum FFF apparatus itself. A single backing pump capable of providing end pressure in the range of 10$^{-3}$ mbar becomes sufficient to implement vacuum-based FFF for processing high-temperature polymers. It offers a novel low-power FFF processing possibility for high-temperature polymers. When comparing the average power needed to maintain the FFF process under vacuum to the process with standard atmospheric conditions, the vacuum-based alternative turns out to be significantly more energy-efficient. In this study the average power necessary to maintain the printing process in a vacuum is in the order of 45 W, while 50 W is needed to maintain the vacuum pressure. For example, 200 W was reported by Hassan et al. [21] for the main phase of the PEEK printing processed on an Apium P220, where no additional heating elements except for the hot end and build plate were in use. When taking into account that most atmospheric FFF printers use convection or radiation-based heating for high-temperature polymer processing, the power needed here is several times higher than it is the case for vacuum FFF.

## Conclusions

This paper provides a quantitative evaluation of how the vacuum environment influences the layer adhesion strength of high-temperature polymers. We conducted a parameter study that shows maximum values of layer adhesion in printed PEEK starting from a pressure of at least 1 $\times 10^{-3}$ mbar. In this study the vacuum environment did not affect the degree of crystallinity of the specimens. However, this may change for different sample geometries. This result further suggests, that since only a single stage vacuum pump is necessary to reach this level of vacuum, vacuum-based FFF printing might become also generally interesting for the processing of high-temperature polymers for a wider range of technical applications than space and medical sectors.

## Figures and Tables

**Fig. 12:**
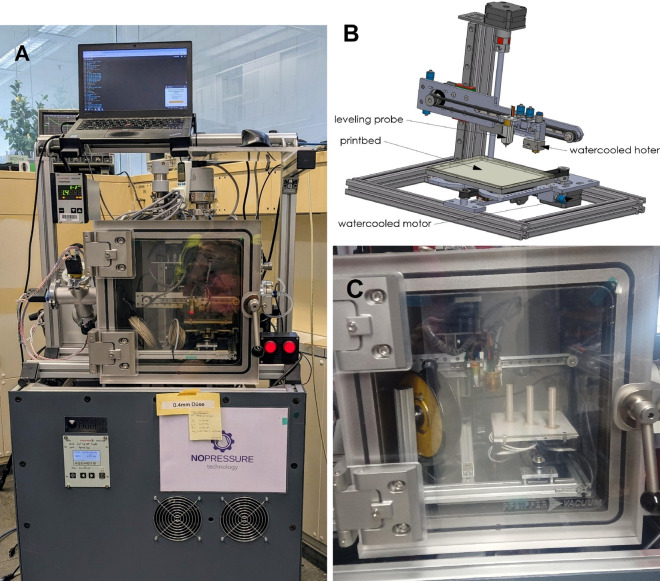
**A** Photograph and **B** CAD model of the vacuum FFF printing device together with **C** a close-up view including the test specimens

## Data Availability

Original data supporting the conclusions of this study were generated using our custom-made vacuum FFF equipment. Derived data that supports the findings of this research can be obtained upon request from the corresponding author.

## Appendix

See Figs. 12, 13.

## References

[1] Estable S, Ahrns I, Regele R, Jankovic M, Brinkmann W, Gancet J, Barrio AM, Leiter P, Colmenero FJ, Ampe A, Ordoubadian B, Chamos A, Caujolle R, Silveira D, Soto I, Shilton M, Vogel T, Bartsch S, Manz M (2023) Outcomes of the period project on in-space manufacturing, assembly and refuelling technologies. J Phys: Conf Ser 2526(1):012121. 10.1088/1742-6596/2526/1/012121

[2] Makaya A, Pambaguian L, Ghidini T, Rohr T, Lafont U, Meurisse A (2022) Towards out of earth manufacturing: overview of the ESA materials and processes activities on manufacturing in space. CEAS Space J 15(1):69–75. 10.1007/s12567-022-00428-1

[3] Kühn-Kauffeldt M, Kühn M, Mallon M, Saur W, Fuchs F (2022) Vacuum arc plasma coating for polymer surface protection—a plasma enhanced in-orbit additive manufacturing concept. Plasma 5(4):470–481. 10.3390/plasma5040035

[4] Navarro SM, Shaikh H, Klop-Packel N, Jethwani H, Wu J, Sutton JP (2021) The impact of additive manufacturing for medical care delivery in space: a systematic review. Adv Mater Technol. 10.1002/admt.202001002

[5] Quinn M, Lafont U, Versteegh J, Guo J (2021) Effect of low vacuum environment on the fused filament fabrication process. CEAS Space J 13(3):369–376. 10.1007/s12567-021-00363-7

[6] Liu T, Zhang M, Kang Y, Tian X, Ding J, Li D (2023) Material extrusion 3d printing of polyether ether ketone in vacuum environment: Heat dissipation mechanism and performance. Addit Manuf 62:103390. 10.1016/j.addma.2023.103390

[7] Spicer R, Miranda F, Cote T, Itchkawich T, Black J (2023) High vacuum capable fused filament fabrication 3d printer, part ii: High-temperature polymers suitable for in-space manufacturing. J Spacecraft Rockets. 10.2514/1.a35709

[8] Spicer R, Miranda F, Cote T, Itchkawich T, Black J (2023) High vacuum capable fused filament fabrication 3d printer, part i: low-temperature polymers and early lessons learned. J Spacecraft Rockets. 10.2514/1.a35708

[9] Seijas M, Piskacev M, Celotti L, Nadalini R, Daurskikh A, Baptista A, Berg M, Caltavituro F, Major I, Devine DM, Maloney A, Lafont U, Makaya A (2024) Closing the loop in space 3d printing: effect of vacuum, recycling, and UV aging on high performance thermoplastics produced via filament extrusion additive manufacturing. Acta Astronautica 219:164–176. 10.1016/j.actaastro.2024.03.015

[10] Zhang J, Van Hooreweder B, Ferraris E (2022) Fused filament fabrication on the moon. JOM 74(3):1111–1119. 10.1007/s11837-021-05031-z

[11] Laurendeau NM (2010) Statistical thermodynamics fundamentals and applications. Cambridge University Press, p 466

[12] Hannoschöck N (2018) Wärmeleitung und -transport: Grundlagen der Wärme- und Stoffübertragung. Springer. 10.1007/978-3-662-57572-7

[13] Coogan TJ, Kazmer DO (2017) Bond and part strength in fused deposition modeling. Rapid Prototyping J 23(2):414–422. 10.1108/rpj-03-2016-0050

[14] Kuznetsov VE, Solonin AN, Tavitov A, Urzhumtsev O, Vakulik A (2020) Increasing strength of FFF three-dimensional printed parts by influencing on temperature-related parameters of the process. Rapid Prototyping J 26(1):107–121. 10.1108/rpj-01-2019-0017

[15] Leguillon D, Martin E, Lafarie-Frenot M-C (2015) Flexural vs. tensile strength in brittle materials. Comptes Rendus Mécanique 343(4):275–281. 10.1016/j.crme.2015.02.003

[16] Evonic Operations GmbH: INFINAM PEEK 9359F (2020). https://www.infinam.com/en/peek-filaments-for-industrial-3d-printing

[17] Arif MF, Kumar S, Varadarajan KM, Cantwell WJ (2018) Performance of biocompatible peek processed by fused deposition additive manufacturing. Mater Design 146:249–259. 10.1016/j.matdes.2018.03.015

[18] Liaw C-Y, Tolbert JW, Chow LW, Guvendiren M (2021) Interlayer bonding strength of 3d printed peek specimens. Soft Matter 17(18):4775–4789. 10.1039/d1sm00417d 33870997 10.1039/d1sm00417d

[19] Doumeng M, Makhlouf L, Berthet F, Marsan O, Delbé K, Denape J, Chabert F (2021) A comparative study of the crystallinity of polyetheretherketone by using density, dsc, xrd, and raman spectroscopy techniques. Polymer Testing 93:106878. 10.1016/j.polymertesting.2020.106878

[20] Doebelin N, Kleeberg R (2015) Profex: a graphical user interface for the Rietveld refinement program BGMN. J Appl Crystallogr 48(5):1573–1580. 10.1107/s1600576715014685 26500466 10.1107/S1600576715014685 PMC4603273

[21] Hassan MR, Jeon HW, Kim G, Park K (2021) The effects of infill patterns and infill percentages on energy consumption in fused filament fabrication using cfr-peek. Rapid Prototyping J 27(10):1886–1899. 10.1108/rpj-11-2020-0288

